# Determining Duration of HER2-Targeted Therapy Using Stem Cell Extinction Models

**DOI:** 10.1371/journal.pone.0046613

**Published:** 2012-12-28

**Authors:** Lindsay Riley, Hua Zhou, Kenneth Lange, Janet S. Sinsheimer, Mary E Sehl

**Affiliations:** 1 Department of Biomathematics, School of Medicine, University of California Los Angeles, Los Angeles, California, United States of America; 2 Department of Statistics, North Carolina State University, Raleigh, North Carolina, United States of America; 3 Department of Human Genetics, School of Medicine, University of California Los Angeles, Los Angeles, California, United States of America; 4 Department of Statistics, University of California Los Angeles, Los Angeles, California, United States of America; 5 Department of Biostatistics, School of Public Health, University of California Los Angeles, Los Angeles, California, United States of America; 6 Division of Hematology-Oncology, Department of Medicine, School of Medicine, University of California Los Angeles, Los Angeles, California, United States of America; University of Chicago, United States of America

## Abstract

**Introduction:**

Trastuzumab dramatically improves survival in breast cancer patients whose tumor overexpresses HER2. A subpopulation of cells in human breast tumors has been identified with characteristics of cancer stem cells. These breast cancer stem-like cells (BCSCs) rely on HER2 signaling for self-renewal, suggesting that HER2-targeted therapy targets BCSCs even when the bulk of the tumor does not overexpress HER2. In order to guide clinical trials examining HER2-targeted therapy in the adjuvant setting, we propose a mathematical model to examine BCSC population dynamics and predict optimal duration of therapy.

**Methods:**

Varying the susceptibility of BCSCs to HER2-targeted therapy, we quantify the average time to extinction of BCSCs. We expand our model using stochastic simulation to include the partially differentiated tumor cells (TCs) that represent bulk tumor population and examine effects of plasticity on required duration of therapy.

**Results:**

Lower susceptibility of BCSCs and increased rates of dedifferentiation entail longer extinction times, indicating a need for prolonged administration of HER2-targeted therapy. We predict that even when therapy does not appreciably reduce tumor size in the advanced cancer setting, it will eventually eradicate the tumor in the adjuvant setting as long as there is at least a modest effect on BCSCs.

**Conclusions:**

We anticipate that our results will inform clinical trials of targeted therapies in planning the duration of therapy needed to eradicate BCSCs. Our predictions also address safety, as longer duration of therapy entails a greater potential impact on normal stem cells that may also be susceptible to stem cell-targeted therapies.

## Introduction

**Figure 1:**
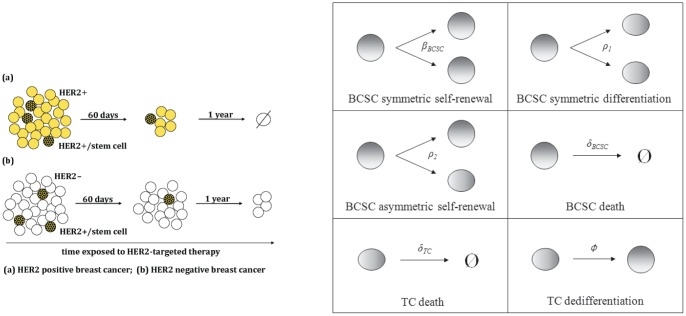
Modeling schematic and event types. Left panel: Schematic representation of modeling question. (a) In HER2-positive tumors, both the breast cancer stem-like cells (BCSCs) and the bulk of the tumor cells (TCs) exhibit increased expression of HER2 and are susceptible to trastuzumab. (b) We hypothesize that in HER2-negative or HER2-weakly expressing tumors, BCSCs express HER2 allowing them to be susceptible to trastuzumab. Right panel: Types of reactions. Breast cancer stem-like cells (BCSCs) can undergo death, self-renewal, and differentiation. Self-renewal can occur either symmetrically or asymmetrically. BCSCs can also symmetrically differentiate, giving rise to two partially differentiated tumor cells (TCs). TCs are born via asymmetric or symmetric differentiation of BCSCs, and can also undergo death. Finally, TCs can dedifferentiate to become stem-like (very rare event).

HER2-targeted therapy combined with standard chemotherapy is known to be effective in treating breast cancer patients in whom the bulk of tumor cells exhibit overexpression of HER2. Among the benefits in the advanced (metastatic) setting are improved survival and increased time to progression [1]. In the adjuvant setting, the addition of trastuzumab to chemotherapy diminishes recurrence rates and improves survival, with relative risk of death cut in half [2]. For this reason, trastuzumab therapy is currently offered to patients whose breast tumor is shown to overexpress HER2 (HER2-positive breast cancer). Recently, a subpopulation of cells in breast tumors has been identified that act like cancer stem cells in their ability to self-renew and recapitulate the heterogeneity of the types of cells in an isolated tumor [3]. Furthermore, these breast cancer stem-like cells (BCSCs) have been shown to express HER2 [4], [5]; HER2 signaling is an important regulator of BCSC self-renewal [4], [6]. These results suggest that the success of trastuzumab therapy in long-term survival originates from its ability to eliminate breast cancer stem-like cells. Targeting BCSCs is an especially attractive strategy in the adjuvant setting, where tumor recurrence is driven by the stem cell population rather than the bulk tumor population [6]–[9]. We hypothesize that even in cases where the bulk of the tumor does not overexpress HER2 (HER2-negative breast cancer), HER2-targeted therapy will eventually eradicate the tumor, provided the therapy is given for a sufficient duration and has a detrimental effect on the life span of BCSCs (see the left panel of Figure 1).

Retrospective analyses of data from a large adjuvant trial of trastuzumab revealed 174 enrolled women with low or normal expression of HER2 [10]. The 82 women randomized to receive a combination of trastuzumab therapy and chemotherapy had improved survival and decreased recurrence compared to the 92 women treated with chemotherapy alone [10]. Based on these preliminary results, future adjuvant studies will investigate whether trastuzumab therapy is indicated in this setting.

We propose a mathematical model to examine the effects of HER2-targeted therapy on breast cancer stem-like cells. Stochastic models are essential to quantify the likelihood of rare events such as extinction. We develop a linear birth-death process model to examine the mean time to extinction of breast cancer stem-like cells (BCSCs) under varying degrees of susceptibility to therapy. Given the assumption that BCSCs are susceptible to HER2-targeted therapy based on the evidence just cited, we calculate the death rate that corresponds to a high likelihood of eradication of BCSCs within 1 year of therapy. We expand the model to include two populations of cells: BCSCs and partially differentiated tumor cells (TCs) representing the bulk of the tumor population. We employ stochastic simulation to examine population dynamics of these cells under trastuzumab therapy, holding the BCSC death rate constant and varying the susceptibility of TCs to therapy. We begin by considering the advanced cancer setting where the bulk tumor (TC) population is large. Here we calculate TC death rates that correspond to two clinical scenarios: 1) HER2-positive breast cancer in which there is a complete or partial response to a short duration (40 days) of HER2-targeted therapy, and 2) HER2-negative breast cancer in which there is no appreciable short-term response to HER2-targeted therapy. Using these death rates as upper and lower bounds for TC response to therapy, we then move to the adjuvant cancer setting, where the tumor cell population size is very small. We predict the duration of therapy necessary to eradicate the entire tumor under varying degrees of TC susceptibility to therapy. Finally, we examine the effects of adding stem cell plasticity (dedifferentiation of partially differentiated cells to stem-like cells) on required duration of therapy.

## Methods

### Mathematical model: Analytic calculation of mean time to extinction

First, we consider cell populations in isolation (either BCSCs or TCs) and apply a birth-death process model to study the dynamics of these populations under a range of death rates corresponding to varying levels of susceptibility to therapy. In this model, a BCSC or TC dies with rate 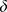 or divides symmetrically with rate 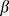. Asymmetric division is addressed in simulation studies of full models presented later, but is ignored presently as it does not change the overall population size of the BCSCs. Symmetric differentiation, giving rise to 2 partially differentiated daughter cells, is accounted for in detailed simulation studies discussed later, but here is handled by simply increasing the death rate of BCSCs.

We have previously employed a theorem from the asymptotic theory of extreme order statistics [11] to study the time to extinction of multiple clans emanating from n stem cells [12], [13]. Briefly, if we can identify two sequences 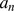 and 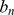 and a function 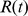 such that 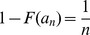 and 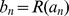, and the ratio of two tail probabilities 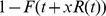 and 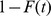 approaches a negative exponential, then we can approximate the shifted distribution 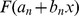 by the Gumbel distribution, which has mean equal to the Euler-Mascheroni constant, and variance equal to 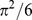. From the a choice of 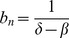, and 
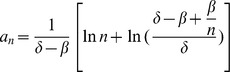
, we arrive at the following equation:
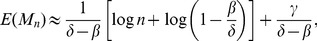
(1)where 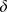 is the death rate per cell, 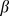 is the birth rate per cell, 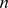 is the initial number of cells, and 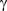 is the Euler-Macheronni constant 0.57722. Here we require the death rate to be greater than the birth rate under therapy. We can also calculate the time that renders the extinction probability equal to a specific value 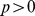 [12] based on the following equation:
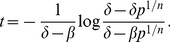
(2)


### Extended model: incorporating TCs and varying degrees of HER2 expression

We now extend the model by letting BCSCs give rise to TCs. This tactic allows us to investigate what is happening to the bulk of the tumor population under therapy. The right panel of Figure 1 depicts the events of our extended model. BCSCs die with rate 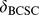 or self-renew by symmetric division, giving rise to two BCSCs with rate 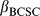, or by asymmetric division, giving rise to one BCSC and one TC with rate 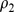. A BCSC can also differentiate symmetrically, giving rise to two TCs with rate 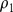. TCs die with rate 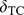.

We employ a fast and accurate approximate stochastic simulation algorithm [14] to study the dynamics of these populations while they are under therapy and experience an increased death rate. We investigate two scenarios. In both cases, BCSCs express HER2 and therefore are susceptible to HER2-targeted therapy. In the first case, we consider TCs that overexpress HER2 and are susceptible to HER2-targeted therapy. In the second case, we consider TCs that do not overexpress HER2 and hence are less susceptible to HER2-targeted therapy. In the second scenario, TCs undergo death closer to the natural rate for partially differentiated tumor cells.

### Parameters and Assumptions

Table 1 lists our choices for parameter estimates used in our stochastic simulations in the adjuvant and advanced cancer settings. For a tumor diameter of 2 cm in the advanced cancer setting, we estimate the total population to be 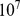, based on population estimates for varying geometries of tumor growth for cells in culture [15]. Based on studies using flow cytometry to identify markers associated with tumorigenicity in a subpopulation of breast cancer cells, we approximate that 1∶100 cancer cells is a breast cancer stem cell [3]. Therefore, we set 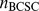 to be 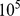 and the remaining cells to belong to the TC population of 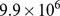. In the adjuvant setting we start with a microscopic foci of disease, approximately 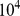 cells with 9,900 TCs and 100 BCSCs.

**Table 1:** Parameter estimates for initial population sizes and event rates.

					Advanced	Adjuvant	
Parameter	Reaction			Symbol	Cancer Setting	Setting	Ref.
# total cells		-		*n*	10,000,000	10,000	[15]
# BCSCs		-		*n*_BCSC_	100,000	100	[3]
# TCs		-		*n*_TC_	9,900,000	9,900	
BCSC symmetric							
self-renewal	BCSC	→	2 BCSC	*β*_BCSC_	0.1 cell^−1^day^−1^	0.2 cell^−1^ day^−1^	
BCSC symmetric							
differentiation	BCSC	→	2 TC	*ρ*_1_	0.1 cell^−1^day^−1^	0.2 cell^−1^day^−1^	
BCSC asymmetric							
division	BCSC	→	BCSC + TC	*ρ*_2_	0.8 cell^−1^day^−1^	1.6 cell^−1^day^−1^	[16], [21], [22]
BCSC death	BCSC	→	0	*δ*_BCSC_	0.13 cell^−1^ day^−1^	0.13 cell ^−1^ day^−1^	
TC death	TC	→	0	*δ*_TC_	0.011 and 0.04 cell^−1^day^−1^	0.011–0.05 cell^−1^day^−1^	[1], [25]
Dedifferentiation							
of TCs	TC	→	BCSC	*φ*	0.0001–0.001 cell^−1^ day^−1^	0.0001–0.001 cell^−1^ day^−1^	

#### Division rates for BCSCs

Under the cancer stem cell hypothesis, we make the assumption that the cells that comprise the bulk tumor population originate from BCSCs, either by asymmetric stem cell division, giving rise to one stem cell and one partially differentiated daughter cell, or by symmetric differentiation, in which one BCSC gives rise to two TCs. BCSCs may also undergo symmetric self-renewal, giving rise to two BCSCs. We make the assumption that TCs do not undergo symmetric division and are solely derived from BCSCs.

We employ the Gompertzian growth estimates to identify birth rate parameters for the bulk of the tumor cells (TCs) in our model. These growth estimates have been previously used to identify mean net growth rate of tumors [16], and are here used as a starting point to identify a birth rate per cell per day for TCs. We describe in detail how these estimates are used as follows. We employ the Gompertzian growth equation [16].
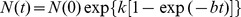
(3)where 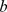 and 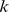 are empirically derived constants, and 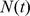 is the number of cells at time 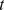. We use parameters 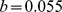 month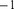 and 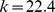, based on observed growth patterns in clinical breast cancer [16]. When the tumor has reached 2 cm (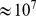 cells), we estimate a period of growth of approximately 2 years. We calculate the growth rate using:
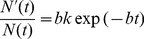
(4)and arrive at a birth rate of 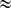 0.01 cell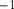 day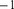 for the bulk TC population. This equates to about 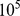 new cells per day. However, under the assumption TCs arise from BCSCs, we need to calculate the rate of TC production per BCSC per day. We thus extrapolate the rate of production through these two mechanisms to be around 1 per stem cell per day. This coincides with an observed cell cycle duration in malignant tumors of approximately 1 day [20]. However, we make the assumption that birth occurs predominantly through the mechanism of asymmetric division of BCSCs (defined as having rate 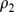), producing one BCSC and one TC, and through symmetric production of 2 TCs from 1 BCSC (defined as having rate 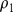). We further assume that asymmetric division is much more common than symmetric self-renewal and symmetric differentiation, based on the references [21]–[23]. Thus we arrive at 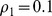 cell
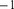
day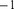, and 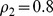 cell
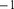
day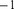. In the adjuvant setting, with a smaller population of 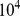 cells, a Gompertzian growth rate estimate of 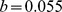 month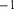 equates to a faster birth rate of 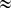 0.02 cell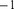 day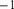 for the bulk TC population.

While normal adult stem cells divide at a slow rate [24], we expect that this rate is increased in cancer stem cells. Based on the assumption that stem-like cells engage in asymmetric division approximately 80% of the time versus self-renewing symmetric division and differentiative symmetric division approximately 20% of the time (10% self-renewing and 10% differentiative) [21], [23], we use a symmetric self-renewal rate of BCSCs of 0.1 cell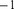 day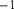.

#### Death rates for BCSCs and TCs

The BCSC death rate is determined so that the mean extinction time of the BCSCs under therapy is 1 year. The assumption that BCSCs are eradicated after 1 year of targeted therapy is based on the previously cited clinical observations of breast cancer [1], [2].

Trastuzumab is a monoclonal antibody that enhances the effects of chemotherapy by providing continuous inhibition to both the extracellular and intracellular domains of the HER2 receptor on breast cancer cells. Normal breast cells express HER2, and in 25% of breast cancers the bulk of the tumor cells overexpress HER2 (HER2-positive breast cancer). Binding to the extracellular domain flags the breast cancer cells for destruction by the immune system, while inhibition of the intracellular domain blocks receptor-mediated intracellular signaling, leading to cell stasis and death [17]–[19]. For these reasons, we have chosen to model the effect of trastuzumab as an increased death rate.

Because HER2-targeted therapy kills bulk tumor cells in the setting of HER2-amplified tumors, we need to model this scenario separately from the case where the bulk tumor cells do not overexpress HER2 (HER2-negative tumors). We make the assumption that the death rate of TC in the HER2 positive setting is much higher than the death rate of TC in the HER2 negative setting. For the first scenario, we extrapolate the death rate from known literature on response rates to HER2-targeted therapy in patients with HER2-positive tumors. A proportion of patients respond to treatment after 2 cycles of therapy (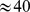 days) [1]. According to the RECIST criteria [25], response is defined by either stable disease, a 30% reduction in the sum of the largest diameter of target lesions compared with baseline (partial response), or disappearance of all target lesiosn (complete response). A 30% reduction size amounts to a 65% reduction in tumor volume [25]. Fitting an exponential rate of decay in tumor population size using the following equation for mean cell count in a birth-death process:
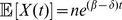
(5)gives a death rate for HER2-positive TCs of approximately 0.05 cell
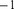
day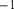. We make the assumption that this rate is much lower for HER2-negative TCs and vary our death rates from 0.011 cell
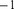
day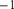 to 0.04 cell
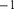
day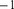. The lower end of the range (0.011 cell
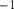
day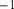) is chosen to be very close to the birth rate of TCs. Thus we calibrate our model to what is observed in the advanced cancer setting and explore what occurs over longer periods of time. We can then predict what might occur in the adjuvant setting, where therapy is administered over the course of a year or a longer period of time.

#### Analytic calculation parameters

Our analytic extinction calculations require the following parameters: initial number of BCSCs and birth and death rates of BCSCs. A separate calculation is run using the initial number of TCs and birth and death rates of TCs. Birth rates are assumed to be 0.2 cell
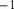
day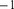 for BCSCs and 0.02 cell
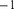
day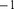 for TCs from Gompertzian growth kinetics. For simplicity, our analytic calculations include separate calculations for the BC population and the TC population, individually modeled as linear birth-death processes. The “birth rate” for TCs developed in the analytic calculation is meant to represent generation of TCs from an underlying BCSC population. The birth rate for TCs differs from our stochastic simulation studies because we no longer incorporate differentiation from stem cells in our model. The birth rate for TCs used in the analytic calculation should match the rate of generation of TCs that is arrived at in the simulation studies. We create a composite birth rate which takes into account birth from symmetric differentiation of BCSCs (multiplied by a factor of 2), asymmetric differentiation of BCSCs, and symmetric division of TCs. The “composite birth rate” is calculated by summing the rates of asymmetric self-renewal and symmetric differentiation of BCSCs and multiplying that number by the initial population size of BCSCs, then dividing by the initial population size of TCs. We vary the death rates of BCSCs and TCs and determine the death rates that ensure that the probability of extinction of both BCSCs and TCs is at least 99% at a year of treatment in the HER2-positive setting. We use these death rates as lower and upper bounds for the range of death rates examined in our stochastic simulation studies.

#### Dedifferentiation rates

Finally we introduce plasticity into our model. The possibility that TCs can dedifferentiate giving rise to BCSCs is an important consideration [26], [27]. We hypothesize that dedifferentiation is a rare event relative to the other events. We therefore examine low rates of conversion from TC to BCSC (0.0001–0.001 cell
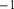
day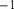).

## Results

### BCSC and TC death rates predict the likelihood that the tumor is eradicated after 1 year of HER2-targeted therapy

Table 2 lists our estimates of mean extinction time for BCSCs and TCs under varying susceptibilities to HER2-targeted therapy in the adjuvant setting (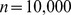). These results are based on direct calculations using equations (1) and (2). In these calculations, we treat the BCSC and TC populations separately. We do not make the assumption that the number of BCSCs remains constant as in previous mathematical models of stem cells but instead assume they are susceptible to therapy. The birth rate of TCs is based on our calculations from the Gompertzian growth model based on a total tumor size of 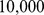 cells. The TC birth rate subsumes birth from symmetric differentiation of BCSCs, asymmetric differentiation of BCSCs, and symmetric division of TC, as described in the Methods section.

**Table 2:** Expected extinction times for BCSCs and TCs under varying susceptibilities to targeted therapy.

BCSC			TC		
Death rate (δ)	Mean Extinction	Prob. of Extinction	Death rate (δ)	Mean Extinction	Prob. of Extinction
(cell^−1^ day^−1^)	Time (SD) (days)	in 1 year	(cell^−1^ day^−1^)	Time (SD) (days)	in 1 year
0.330	33 (10)	>0.9999	0.081	156 (21)	>0.9999
0.233	100 (39)	0.9999	0.071	186 (25)	0.9999
0.224	125 (53)	0.9985	0.061	230 (32)	0.9977
0.216	171 (82)	0.9768	0.052	289 (40)	0.9525
0.213	196 (100)	0.9459	0.049	316 (44)	0.8754
0.201	232 (128)	0.8810	0.044	389 (54)	0.3764
0.207	289 (180)	0.7606	0.041	439 (62)	0.0736
0.204	397 (299)	0.5753	0.035	595 (86)	<0.0001
0.203	508 (449)	0.4663	0.029	929 (138)	<0.0001
0.202	630 (641)	0.4002	0.026	1385 (214)	<0.0001
0.201	978 (1283)	0.3256	0.021	8979 (1796)	<0.0001

As we decrease the susceptibility to therapy, the mean extinction time lengthens. We also examine the probability of extinction in 1 year, the typical period of administration of trastuzumab in the adjuvant setting. If one wished to be at least 99% sure that BCSCs are eradicated after a year, the death rate for BCSCs under trastuzumab should be 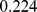 cell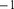 day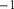 starting with microscopic disease. Likewise, to be 99% sure that all TCs are eradicated after a year, the death rate for TCs under trastuzumab should be at least 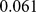 cell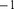 day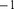.

### Effects of HER2-targeted therapy are not always observed in the advanced cancer setting

**Figure 2:**
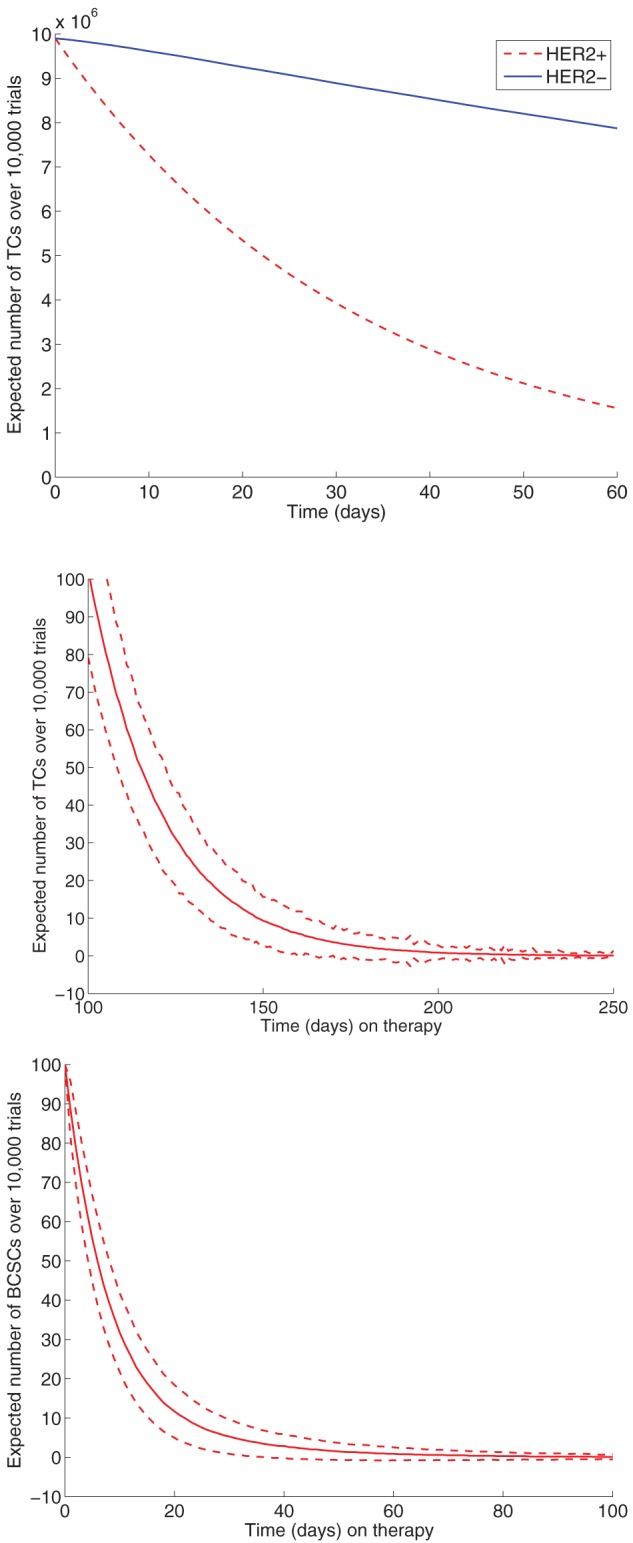
Average trajectories of TCs over time under therapy in the advanced cancer setting (HER2-positive and HER2-negative). During the first 2 cycles of therapy, we observe a decline in BCSCs. In the HER2-positive case where the bulk of the tumor population overexpresses HER2 (corresponding to a death rate of 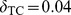 cell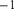 day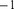), the TC population declines rapidly over the first 40 days. When TCs do not overexpress HER2 (HER2-negative case, corresponding to a death rate of 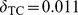 cell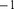 day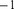), there is not a visible difference in tumor size.

The top panel of Figure 2 reveals results from stochastic simulation predicting the average trajectories for TCs under therapy. We consider here the advanced cancer setting, where we start with a 2 cm tumor (number of total cells 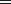
10,000,000) and examine tumor size after 2 cycles of therapy, usually after about 6 weeks. A TC death rate of 0.05 corresponds to a substantial decline in the bulk tumor population by 6 weeks, corresponding to an observed response to therapy. Thus we suggest 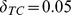 as an appropriate death rate for TCs susceptible to HER2-targeted therapy in the HER2-positive setting. In contrast, when we take 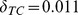, there is a less appreciable decline in the TC count. This death rate corresponds to the HER2-negative setting, where the bulk of the tumor population does not overexpress HER2, consistent with the negligible changes seen in tumor imaging.

### HER2-positive and HER2-negative tumors are driven to extinction after a sufficient duration of therapy in the adjuvant setting

Using the postulated death rate parameters corresponding to HER2-negative and HER2-positive disease, we can examine the fate of BCSC and TC populations in the adjuvant setting using stochastic simulation. Because total tumor size is smaller (10,000 cells), the birth rates for BCSCs and TCs are accordingly increased to follow Gomperzian tumor growth patterns. Middle and bottom panels of Figure 2 reveal the average number of BCSCs and TCs over time under therapy, in the HER2-positive scenario where the bulk of the tumor population is susceptible to therapy (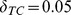). While the mean trajectories for both BCSCs and TCs both decay over time, there is substantial variation in population dynamics as displayed by the standard deviation plotted in this figure.

**Figure 3:**
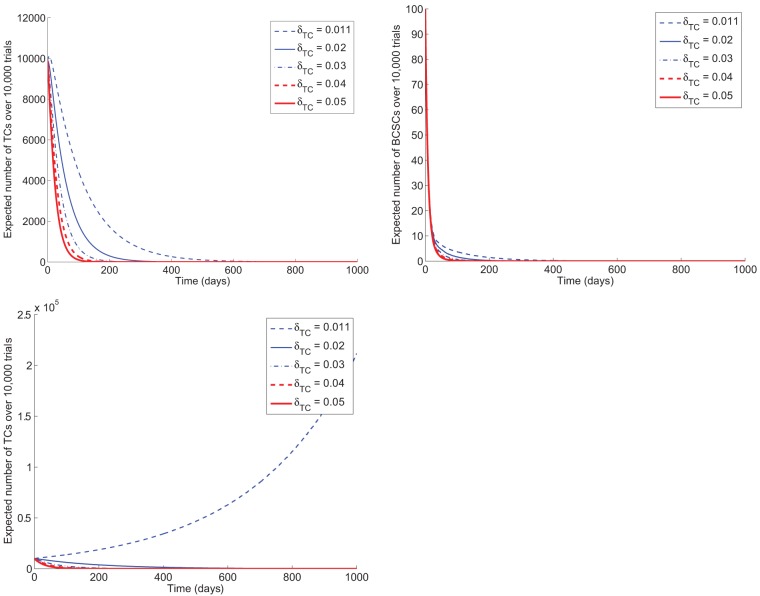
Response to therapy in the adjuvant setting. Average trajectories of TCs (left panels) and BCSCs (right panel) over 1 year of therapy in the adjuvant setting, under varying TC death rates (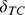) and dedifferentiation rates. In all cases, the death rate for BCSCs is 0.23 cell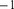 day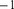. In top panels, dedifferentiation is rare (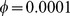 cell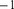 day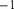). The HER2-positive setting corresponds to a TC death rate (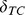) of 0.04–0.05 cell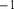 day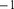, whereas in the HER2-negative setting, 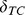 is nearly equal to the TC birth rate. Even when 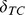 is minimal (HER2-negative), the tumor is eventually driven extinct in the adjuvant setting, though it may take more than a year of therapy. The bottom panel examines the effects of plasticity. If we consider a fast rate of dedifferentiation of TCs (0.001 cell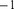 day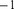), we find that it will take considerably longer than a year to eradicate the tumor when TCs are less sensitive to therapy. In the case where the TCs are insensitive to therapy and the dedifferentiation rate is sufficiently high, the tumor will grow.

Figure 3 shows trajectories of BCSCs and TCs, respectively, under 1 year of therapy, while varying susceptibility of TCs to therapy. Death rates for TCs correspond to varying degrees of susceptibility of the bulk of the tumor population to HER2-targeted therapy. The range of rates (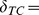
0.011 to 0.05) is derived from the upper and lower bounds corresponding to HER2-positive and HER2-negative settings derived from results modeling the advanced cancer setting. In all cases, both BCSC and TC populations are driven to extinction. In accord with our assumptions, BCSCs die under therapy at approximately the same rate in each scenario. The lower panel shows the BCSC population approaching extinction in less than 1 year.

When the bulk of the tumor population overexpresses HER2, the tumor population is driven extinct fairly early (less than 200 days). However, when the death rate of TCs approaches its birth rate, the time to extinction for TCs can be more than a year. This scenario corresponds to the case where TCs are insensitive to HER2-targeted therapy, but slowly die out because the stem cell-like population is being targeted. One can see from these figures that even in this case, the TCs will eventually reach extinction.

When we examine the trajectories of BCSCs and TCs under a longer period of exposure to HER2-targeted therapy, we see that eventually in all cases, the TCs are driven extinct (see top panels of Figure 3). After the BCSC population goes extinct, around 70 days, the TC population begins to drop and eventually dies out. When we consider the HER2-negative case in which TCs are relatively insensitive to HER2-targeted therapy, we can estimate how long it will take for the TCs to die off once the BCSC pool is depleted. In Figure 3, we can see that even in cases where the bulk of the tumor is insensitive to HER2-targeted therapy, if the BCSCs are susceptible, the tumor will reach extinction in the adjuvant setting after 2–3 years of therapy. Specifically, when we set 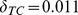, we calculate the mean extinction time for BCSCs to be 77.3 days 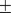
36.6 (mean 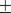 SD), and the mean extinction time for TCs to be 1097.9 days 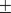 148.7 (mean 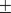 SD) 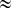 3 years.

### Prolonged duration of therapy is required for higher rates of TC dedifferentiation

Finally, we examine the effects of plasticity. In previous simulations, the event of dedifferentiation was very rare (rate of conversion of TCs to BCSCs was 0.0001 cell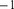 day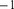). We can also investigate the case where the event of dedifferentiation is more common. When we increase this rate to 0.001 cell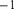 day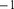 (see bottom panel of Figure 3), we see that the TC population is still driven to extinction in most cases. However, if the TCs are completely insensitive to therapy, the tumor population will grow exponentially if there is a sufficient amount of plasticity in the model (high rate of dedifferentiation of TCs to BCSCs). These results highlight the importance of targeting both the TC and BCSC populations simultaneously.

## Discussion

Our predictions suggest that therapies that target breast cancer stem-like cells may be advantageous in the adjuvant setting, even when very little or no change is observed in the advanced cancer setting. Therapies that target HER2 signaling may be effective in eliminating small populations of breast cancer stem-like cells in cases where HER2 is not overexpressed by the bulk of the tumor population. To eradicate all of the tumor cells in HER2-negative breast cancer, it may be necessary to treat for longer periods of time than is conventional for adjuvant trials. Our results, along with the work of others [28], [29], show the importance of mathematical modeling in informing therapeutic decisions in breast cancer.

Our findings highlight the importance of tracking the effect of therapy on the subpopulation of tumor cells that is capable of self-renewal and recapitulating the tumor population. Traditional criteria tracking the effectiveness of a therapy focus on the bulk of the tumor population. If the stem-like cell population is targeted, it may take long periods of time to observe significant effects on the bulk population, but these effects may eventually lead to eradication of the tumor. As technologies are developed to identify and track the stem-like cells in a population, death rates for this subpopulation of cells will be better estimated. Our models could then be re-employed to predict the duration of therapy needed to eradicate this population and the entire tumor.

Plasticity is now recognized as an important aspect of cancer stem cell dynamics [26], [27]. We incorporate dedifferentiation of tumor cells to stem-like cells in our models and hypothesize that this is a rare event. Under this scenario, we show that a complete response is feasible by targeting the stem-like cell population alone. In reality, stem cell targeted therapies will likely be combined with cytotoxic chemotherapy. Further models could incorporate higher rates of dedifferentiation and the evaluation of combinations of therapies that simultaneously target a rapidly dividing BCSC subpopulation and progenitor cells with limited self-renewal potential and clonogenic cell death. Finally, future models should address the critical issue of a quiescent BCSC subpopulation that may be less sensitive to therapy and lead to tumor recurrence.

Experimental determination of the birth rates and death rates for both partially differentiated cells and stem-like cells would increase the precision of our results. Our predictions are based on the difference between death and birth rates rather than the rates themselves, and we provide a bound on the range of possible observed effects with therapy. Holding death rates of BCSCs constant and varying the symmetric self-renewal rate would produce similar results to what we have found. Decreasing asymmetric self-renewal rates would accelerate decline in the TC population leading to earlier extinction times. Furthermore, the dependence of extinction times on the difference between birth and death rates is stronger than the dependence of extinction times on initial BCSC population size. Thus, we do not expect a dramatic change in our qualitative results to be achieved by varying the initial proportion of BCSCs in the tumor. As stem cell targeted therapies are developed and birth and death rates become known, our models will be helpful in conjunction with experiment in predicting the efficacy of proposed combinations of therapies targeting ErbB receptors. More sophisticated models might incorporate limited self-renewal of the TC population and clonogenic cell death. However based on the available experimental data and our simulations, we do not expect predictions from a model incorporating these aspects to be substantially different from ours. Other limitations of our work include the absence of microenvironmental, immunological, and spatial effects in modeling drug delivery to a solid tumor. We assume a well-mixed system in which cells within a given population type are equally susceptible to a given therapy. Incorporating these effects will require knowledge of how breast cancer stem-like cells are distributed within a tumor. Future models should address the critical issues of tumor architecture that usually limits proliferation to the outer rim, and a quiescent BCSC subpopulation that may be less sensitive to therapy, eventually leading to tumor recurrence. Models accounting for the quiescent stem cell population would require consideration of the event of reawakening and entail longer predicted durations of therapy. Finally, to fully undertand breast cancer stem cell dynamics, further progress must be made in understanding and modeling normal breast stem cell biology, both during development and in adult stem cells.

Our model has both stochastic and deterministic components. While the average trajectories of BCSCs and TCs appear to follow a deterministic decay under therapy, there is substantial variation in population counts, especially as the populations approach extinction. In order to calculate mean extinction times, it is essential to employ a probabilistic model.

Findings from retrospective review of adjuvant clinical trials have shown that women with HER2-negative breast cancer may benefit from therapy targeting HER2. Our results lend support to the hypothesis that this benefit may be caused by the ability of HER2-targeted therapies to interrupt signaling in breast cancer stem-like cells. We show that the addition of HER2-targeted therapy to chemotherapy may lead to cure in the adjuvant setting even when the bulk of the tumor population removed does not overexpress HER2. We are confident that our predictions, combined with experimentally derived birth and death rates, will be a useful tool to optimize therapy design and duration.
